# Steatosis and Steatohepatitis: Complex Disorders

**DOI:** 10.3390/ijms15069924

**Published:** 2014-06-03

**Authors:** Kira Bettermann, Tabea Hohensee, Johannes Haybaeck

**Affiliations:** Institute of Pathology, Medical University of Graz, Auenbruggerplatz 25, Graz A-8036, Austria; E-Mails: kira.bettermann@gmx.de (K.B.); tabeahohensee87@gmail.com (T.H.)

**Keywords:** steatosis, steatohepatitis, ASH, NASH

## Abstract

Non-alcoholic fatty liver disease (NAFLD) which includes steatosis and steatohepatitis, in particular non-alcoholic steatohepatitis (NASH), is a rising health problem world-wide and should be separated from alcoholic steatohepatitis (ASH). NAFLD is regarded as hepatic manifestation of the metabolic syndrome (MetSy), being tightly linked to obesity and type 2 diabetes mellitus (T2DM). Development of steatosis, liver fibrosis and cirrhosis often progresses towards hepatocellular carcinogenesis and frequently results in the indication for liver transplantation, underlining the clinical significance of this disease complex. Work on different murine models and several human patients studies led to the identification of different molecular key players as well as epigenetic factors like miRNAs and SNPs, which have a promoting or protecting function in AFLD/ASH or NAFLD/NASH. To which extent they might be translated into human biology and pathogenesis is still questionable and needs further investigation regarding diagnostic parameters, drug development and a better understanding of the genetic impact. In this review we give an overview about the currently available knowledge and recent findings regarding the development and progression of this disease.

## 1. Introduction

Non-alcoholic fatty liver disease (NAFLD) was first described in 1980 and has recently become one of the most prevalent liver diseases in developing as well as in developed countries [1,2,3,4,5]. NAFLD represents a spectrum ranging from simple steatosis to hepatic inflammation, hepatocyte ballooning, presence of Mallory-Denk bodies (MDBs) and fibrosis, which is referred as non-alcoholic steatohepatitis (NASH) in case of proven alcohol abstinence [6,7,8,9,10]. The distinction between these diseases is important as steatosis is less likely to evolve into severe liver related complications [11,12], whereas NASH might progress to liver cirrhosis and hepatocellular carcinoma (HCC) [8,12].

NAFLD is strongly associated with obesity, which is frequently accompanied by the metabolic syndrome (MetS), reduced glucose tolerance and type 2 diabetes mellitus (T2DM) [13,14].

A well-established theory says that the development from steatosis to NASH follows a “two-hit” concept where the first hit is the sensitization of the liver to injurious effects and the second one the cause of inflammation and fibrosis [15]. However, this theory has been put into question based on insights on the interaction between insulin resistance, adipose tissue inflammation and adipokines. Therefore a modification, the “multiple-parallel-hit” hypothesis has arisen [16], meaning that after the first hit, which is insulin resistance in combination with many associated metabolic dysfunctions the liver is at risk for other hits resulting in NASH and liver fibrosis. Both hypotheses have in common that simple steatosis is believed to be the basis for the development of NASH [17].

## 2. Epidemiology and Risk Factors

Many studies have been published from developed nations like the United States, Europe and Australia, which show a prevalence of NASH in 20% to 30% of the population [18,19,20,21]. Asia, which was commonly thought to be a low risk area shows a prevalence range between 15% and 30% [22,23,24]. In one study from Korea 51% of the tested patients suffered from NAFLD [25].

Demographic analysis indicates how ethnicity influences the extent of NASH incidence. In Hispanic populations the prevalence of NAFLD and NASH is the most common followed by Non-Hispanic Whites and African Americans [21,26,27]. The lowest rate of NAFLD reported was in American Indian and Alaska-Native populations, which ranges from 0.4% to 2% [28,29]. Children from these ethnicities are predisposed in a similar way. The highest incidence was observed in Hispanics and Asians [30,31], but up to now it is not known how far the genetics and racial differences compared to the environmental factors reflect the incidence of NAFLD.

A clear gender difference concerning amount and distribution of body fat is also recognized. Men are more likely to develop NAFLD based on their tendency to store fat around organs in the abdominal cavity [32]. One Asian study showed on 26,527 patients that the prevalence of NAFLD was 31% in men compared to 13% in women [33]. A study from India demonstrated that the majority of those with NAFLD are men [34]. These results may correlate with the elevated aminotransferase levels and the presence of NASH and hepatic fibrosis in males [26,35].

One reason for this inequality in fat distribution is the twice as high hepatic lipase activity in men, which may contribute to a lower high density lipoprotein (HDL) cholesterol level and heightened levels of dense low density lipoprotein (LDL) particles [36]. This distinction in hepatic lipase activity can be traced back to the suppression of androgenic steroids [37]. In addition, it has been shown that female steroids, especially estrogen, promote the utilization of fat as an energy source, as well the non-visceral fat, which makes women less likely to accumulate abdominal fat [38,39,40].

There are several risk factors known which are likely to be required for the development of obesity, visceral adiposity, insulin resistance, steatosis and fibrosis: genetic predisposition, ethnicity, age, gender and lifestyle. Most patients diagnosed with NAFLD have these metabolic risk factors. In 57% to 98% [41,42,43] of overweight people NAFLD is diagnosed, whereas simple steatosis ranges between 30% and 37% [44,45]. Compared to non-obese patients, being overweight increases the risk to develop fibrosis three-fold [13]. Especially visceral obesity shows a higher correlation to fatty liver than normal body mass. This increased fat accumulation in the abdomen causes higher lipolytic activity and thus increased plasma free fatty acid (FFA) levels [46,47] that are toxic for hepatocytes and mitochondria, leading to apoptosis and inflammation.

Insulin resistance is an indicator of the risk to develop NAFLD [44]. Recently, several studies have shown that adipose tissue insulin resistance (Adipo-IR) could be a predictor of the histological changes of the liver and, for patients already suffering from NAFLD, a possible predictor of fibrosis progression [48,49].

## 3. Genomics and Epigenomics

Several molecular mechanisms are believed to be associated with the pathogenesis of NASH, but their influence on NAFLD still needs to be determined [50,51]. The hedgehog (hh) pathway plays a critical role in the immune response [52] and is involved in natural killer T cell enrichment leading to a fibrogenetic hepatic response, which is observed in NASH [53,54]. miRNAs, DNA methylation patterns, histone modifications and ubiquitination have therefore been investigated. miRNAs are proven to be important regulators of cellular processes [55,56]. In a recent study, 46 out of 474 miRNAs were differentially expressed in NASH patients compared to a control group [57]. The most under-expressed one was miRNA-122, being involved in lipid and cholesterol metabolism as well as in adipocyte differentiation. Further, an up-regulated expression level of miRNA-335 was determined in murine liver and white adipose tissue. miRNA-335 is involved in increased body, liver and white adipose tissue weight and in heightened cholesterol- and hepatic triglyceride levels [58].

Also the miRNA-34 family, being a direct transcriptional target of p53, is dysregulated in NAFLD [59]. Especially miRNA-34a expression, is involved in apoptosis, increases in correlation to the severity of disease [60]. NAFLD appears to be multifactorial, including environmental as well as genetic factors [27]. There are several genetic variants known to be involved in energy balance. The most prominent one is rs738409, a SNP in the *patatin-like phospholipase domain-containing 3* (*PNPLA3*) gene [61]. PNPLA3 is a membrane-bound protein with lypolytic and lipogenic activities being expressed in hepatocytes and adipocytes [62,63]. Other genetic variants being associated with NAFLD involve genes related to oxidative stress, inflammation and fibrogenesis, such as *super oxide dismutase 2 (SOD2)* [64]. Controversially, even with a proven association of *PNPLA3* with NAFLD [62,63], there is no effect on very low density lipoprotein (VLDL), LDL, HDL, insulin resistance, total and circulating cholesterol levels, which are accounted as primary risk factors [65,66].

The *PNPLA3* genotype distribution was reported to differ between patients affected by NAFLD and NASH. This indicates, that NASH might genetically differ from the other spectrum of the disease complex, thus *PNPLA3* being associated with hepatic necroinflammation [61].

## 4. Diagnosis

Several diagnostic methods are available to evaluate NAFLD. The liver biopsy remains the gold standard, but as it is invasive, it cannot be used for population-based studies. Thus, several non-invasive methods have been introduced including imaging techniques like magnetic resonance imaging (MRI) and the measurement of serum markers such as alanine aminotransferase (ALT) and aspartate aminotransferase (AST).

In 45%–100% of NAFLD cases the disorder remains asymptomatic [11] and 55%–79% of NAFLD patients have inconspicuous transaminase levels [67]. Therefore aminotransferases in combination with the measurement of glucose, triglyceride, cholesterol and lipoprotein serum levels are used in combination with anthropometric parameters such as body mass index (BMI) and fat distribution and other information, like gender, age, lifestyle habits and family disease history [2,68].

Liver ultrasonography is a good tool to estimate the degree and extent of steatosis by using a series of ultrasonographical characteristics [69]. Computed tomography (CT) is more specific to ultrasonography, but because of the radiation exposure it is only used for research studies in adults. An alternative would be magnetic resonance tomography (MRT), which is currently tested with different phase-shift imaging methods to heighten the accuracy of the total liver-fat content measurement [70,71,72], but due to the high costs it remains primarily investigational. Transient elastography (TE) is an ultra-sound based method to evaluate fibrosis. Recently, a study demonstrated that TE is a valuable tool for the detection and staging of fibroses even in children [73]. Another study showed that TE has a sensitivity of 91% and a specificity of 75% in detecting stage 3 or higher fibrosis in NAFLD patients [2], but here the failure rate increases in correlation to body weight as well. An improvement of this technique would be magnetic resonance elastography, measuring the stiffness of the entire organ, in contrast to TE, which measures only around 50 cm^2^ [74].

Another promising technique to predict fibrosis in NAFLD patients are the FibroTest (FT) and the Actitest (Biopredictive Paris, France, FibroSURE in the US patented artificial intelligence algorithm USPTO 6,631,330). In combination with the fibrosis index which includes a various amount of markers, like alanine aminotransferase (ALT), apolipoprotein A1, α2-macroglobulin (A2M) and others, this combination showed a sensitivity of 77% and a specificity of 98% by diagnosing fibrosis in 170 NAFLD patients [75].

In summary, liver biopsy remains the gold standard to characterize liver diseases. It is currently the only tool to assess the degree of inflammation as well the degree and stage of fibrosis, but the appearance of new promising diagnostic techniques such as the FT, the Actitest or magnetic resonance elastography, will hopefully replace usage of liver biopsy.

## 5. Molecular Pathology

Progression of steatosis is mainly mediated by cytokines and a panel of different enzymes which fulfill important roles in lipogenesis and lipolysis [76,77,78].

In this context, visceral fat seems to play a role in NAFLD progression due to its ability to actively secret a multitude of different adipokines such as tumor necrosis factor alpha (TNFα), interleukin-6 (IL-6), macrophage chemoattractant protein (MCP)-1 and resistin, which promote insulin resistance and type 2 diabetes mellitus (TD2M) [79,80,81]. Obesity and increased levels of inflammatory cytokines are well established as tightly related to each other [82,83].

Different studies on obese patients and obese murine models showed a correlation between visceral fat, insulin resistance and an increased predisposition for NAFLD and NASH [84,85,86]. The protein hormone adiponectin and the cytokine TNFα have extremely important functions in this disease. Adiponectin is synthesized by visceral adipocytes and plays an important role regarding insulin tolerance, because it directly activates 5'-adenosine monophosphate-activated protein kinase (AMPK), glucose consumption and fatty acid oxidation [87]. Measurement of adiponectin serum levels from visceral adipose subjects of different ages revealed significantly decreased levels of adiponectin representing an inverse relation to the rate of body fat [88,89,90]. The reduction of adiponectin seems to have a major impact on the development of hepatic steatosis and NASH caused by its direct antagonistic effect on TNFα, one of the most important cytokines in mediating inflammation. As opposed to this, human NAFLD patients have been demonstrated to display increased serum levels of TNFα [89,91]. It appears that the ratio between adiponectin and TNFα might be essential for the progression of NASH [92].

TNFα functions antagonistically to adiponectin and is secreted by hepatocytes and adipocytes. As a master regulator of inflammation, TNFα is involved in both regulation of other cytokines like IL-6 and in the NF-κB signaling cascade [93,94].

## 6. Treatment and Prevention

Because of the incomplete understanding of the molecular pathogenesis of NAFLD, the current therapy focuses rather on preventing risk factors or treatment of side effects accompanying obesity. These interventions target lifestyle changes, including diet and physical exercises mainly in combination with pharmacotherapy. This includes next to NAFLD treatment, improvement of the MetS, T2DM and related cardiovascular diseases. The most important lifestyle modification is weight loss, which has been shown to improve serum aminotransferases and BMI, but also a decrease in hepatic steatosis [95,96,97]. One study of biopsy-proven NASH patients had shown that patients who lost 5% of their body weight had improved insulin sensitivity and steatosis. Moreover, it could be demonstrated that only a weight reduction of 9% leads to significant positive changes concerning inflammation, ballooning and the NAFLD activity score [98]. However, rapid weight loss (24% body weight in 8 weeks) increases portal inflammation and fibrosis as well [99].

An important mediator for hepatic injury in NASH is oxidative stress [100,101,102], therefore an antioxidant therapy may slow the progression of steatosis and NASH. A prominent antioxidant is Vitamin E which when used in dosages ranging from 400 to 1200 units per day over a time period of 2–4 months leads to a normalization of obese children [103]. The Pioglitazone, Vitamin E, or Placebo for Nonalcoholic Steatohepatitis (PIVENS) trial, the largest study evaluating the effect of Vitamin E in nondiabetic adults with NASH demonstrated a significant clinical and biochemical improvement [104].

Metformin which can also applied in combination with Pioglitazone reduces glucose production and increases insulin sensitivity in patients suffering from T2DM [105,106], but subsequent randomized clinical trials were not able to proof significant differences in liver histology [107,108]. Therefore metformin as therapy for NASH patients is not recommended.

Thiazolidinediones (TZDs) increase hepatic insulin sensitivity and improve glucose and lipid utilization in T2DM [105]. One study with 22 NASH patients showed improved inflammation, ballooning and fibrosis, but 67% of the patients gained weight [109]. Another double-blind study recognized a significant improved steatosis, ballooning and inflammation in 73% of the NASH patients [110].

Long-chain omega-3 fatty acids showed a putative decrease in steatosis and markers of inflammation, and improvement of insulin sensitivity in experimental studies [111]. Pentoxifylline, a xanthine derivative, and promising in several studies, could be worth for further testing [112]. At this time it should be concluded that there is no drug treatment available that is proven to cure fatty liver without side effects.

## 7. Murine Non-Alcoholic Fatty Liver Disease (NAFLD) Models and Their Potential Human Relevance

Inhibitor of nuclear factor kappa-B kinase subunit beta (IKKβ) and nuclear factor kappa-light-chain-enhancer of activated B cells (NF-κB) have been shown to be crucial in promoting inflammation and insulin resistance in different studies investigating murine transgenic and knockout models fed with high fat diet (HFD) [113,114,115]. The use of the IKKβ blocker aspirin or of other salicylates had a markedly positive effect on insulin resistance in patients with TD2M as well as in insulin resistant mouse models [113]. Generally, salicylates seem to have a potentially positive influence on the development of NASH and its concomitant effects. Therefore, it would be certainly a useful drug, but the side effects of salicylates such as liver toxicity and anticoagulation have to be seen critical and are currently under debate.

Besides TNFα, IL-6 is another important adipocytokine linked to NAFLD and obesity. Similarly to TNFα, enhanced amounts of IL-6 are also secreted by the visceral fat of obese subjects [81]. In the work by Cai *et al.* [115] increased levels of IL-6 in NAFLD patients were associated with IKKβ mediated NF-κB activation. This study demonstrated a significantly positive influence of an anti-IL-6 antibody on the progression of insulin resistance [115]. Overall, HFD leads to a considerable activation of TNFα and IL-6. Nevertheless, inhibition of TNFα via TNFR1 or of IL-6 through antibodies or blocking of IKKβ activity protects against hepatic steatosis. Moreover, ablation of Fas (CD95), a further member of the tumor necrosis factor receptor (TNFR)-superfamily, in murine adipocytes has a similar positive effect on insulin resistance during HFD as demonstrated for TNFα and IL-6 [116].

One trigger of inflammation is caused by endotoxins/lipopolysaccharides (LPS) from gut bacteria. LPS is one element of the outer membrane of gram-negative bacteria. It is released into the gut and circulates through the portal blood directly into the liver [78]. There, LPS activates Toll-like receptor 4 (TLR 4), a member of the interleukin-1 receptor/toll-like receptor superfamily. TLRs are located in the liver on the membrane of Kupffer cells, hepatocytes, hepatic stellate cells, biliary epithelial cells, hepatic dendritic cells and liver sinusoidal epithelial cells [117]. The work by Cani and colleagues showed that LPS has the potential to promote insulin resistance. The authors set C57Bl6/J mice on a HFD for 4 weeks and measured 2–3 times higher LPS blood plasma concentrations compared to normal chow fed mice. An increased growth rate of LPS-releasing microbiota in the gut was reported. Interestingly, similar results were generated upon administration of subcutaneously injected LPS (“induction of endotoxemia”) for 4 weeks [118]. Other murine models have validated that NASH induced by high fructose or methionine-choline deficient (MCD) diet also leads to increased expression rates of *Tlr4* [119]. Conversely, deletion of *Tlr4* in mice by depletion of Kupffer cells through chlodronate administration, or generated by a single point mutation fed with MCD diet, showed less hepatic steatosis in comparison to the wild-type controls [120]. These results give evidence that TLR4 and Kupffer cells might have a critical function in mediating steatohepatitis and other concomitant effects like fibrosis. It is under debate if downstream effector molecules such as reactive oxygen species (ROS), chaperone proteins or transcription factors could also play a role in NASH development. Next to the study by Cani, Yoshimoto and colleagues [121] recently published that the gut microbiota has a great impact on HCC development in case of obesity. Genetic obesity seems to directly influence the composition of gut microbiota, thus promoting enhanced secretion of the metabolite deoxycholic acid (DCA), which has DNA damaging properties. Increased levels of DCA stimulate hepatic stellate cells (HSCs) to release proinflammatory and tumor-promoting factors through the liver. In their obesity mouse model, application of a cancer-promoting chemical in these mice increased their susceptibility to develop HCC. Similar results such as activated HSCs in the vicinity of HCCs could be obtained in human NASH patients, underlining the impact of obesity and gut bacteria composition [121].

**Figure 1:**
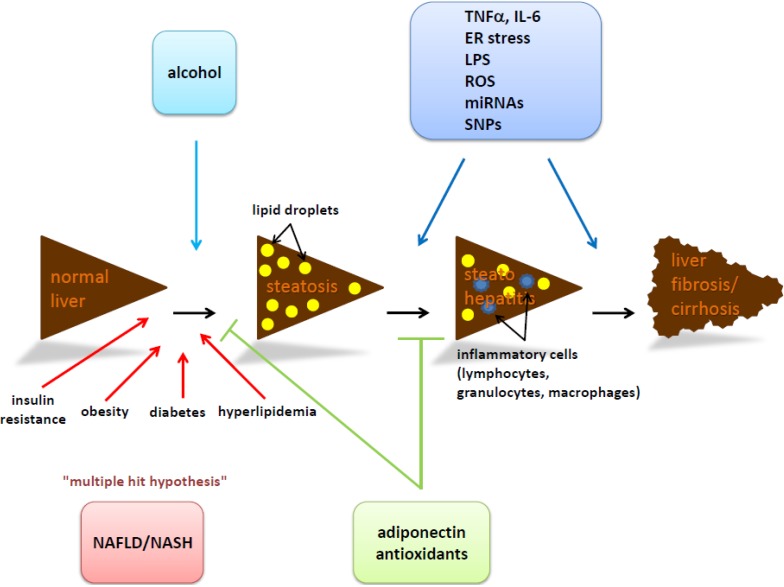
Schematic illustration of non-alcoholic fatty liver disease (NAFLD)/non-alcoholic steatohepatitis (NASH) progression. A lifestyle with a balanced diet, moderate alcohol consumption and regular exercise supports the maintenance of an intact liver function. The intake of high energy food, less exercise and/or enhanced drinking of alcohol (light blue rectangle and arrow) leads to increased amounts of triglycerides (TG) in the liver which are stored as lipid droplets (yellow dots). Over time the degree of fatty change might reach an extent of hepatic steatosis. Concomitant effects like insulin resistance, obesity, type 2 diabetes mellitus (T2DM) and hyperlipidemia have a promoting effect (red arrows). It is under debate if the development of steatosis and NAFLD/NASH is the result of a “two” or “multiple hit event’’ (red rectangle). Adipocytokines, endoplasmic reticulum (ER) stress, endotoxins from gut bacteria, reactive oxygen species (ROS) and epigenetic modifications (dark blue rectangle and arrows) are potential mediators promoting the development from hepatic steatosis to steatohepatitis and liver fibrosis and/or cirrhosis. Under physiological conditions fatty acid metabolism is tightly controlled by antagonists such as adiponectin and antioxidants (green rectangle and arrows) which are involved in insulin tolerance, glucose consumption and fatty acid oxidation as well as in elimination of ROS.

Based on the results of various mouse models and the medical diagnosis of patients, it is debated if NASH/NAFLD is the result of multiple hit events (Figure 1) [119]. The interplay of oxidative stress and increased cytokine release and enhanced hepatocyte death are mainly responsible for this disease. In the course of NASH, several signaling pathways are activated which leads to cell death such as apoptosis, autophagy, necrosis, necroptosis and maybe pyroptosis [119].

A main feature of hepatic steatosis is an unbalanced accumulation of triglycerides (TG). If the energy intake is higher than the usage, an increased storage of TG in the liver has to follow. Several proteins, such as fatty acid transport proteins (FATPs), fatty acid translocase (also known as CD36) and fatty acid binding proteins (FABPs) regulate the uptake of fatty acids into hepatocytes [122].

A second promoter of steatohepatitis is an increased synthesis of fatty acids. Hyperinsulinemia is the main driver for *de novo* lipogenesis and is often an accompanying symptom of NAFLD. Lipogenesis is regulated by transcription factors which are initiated by insulin such as sterol regulatory element binding protein (SREBP)-1c [123]. SREBP-1c controls transcription of other important proteins, which are associated with lipogenesis and TG synthesis, e.g., fatty acid synthase (FAS), stearoyl-CoA desaturase (SCD) 1 and acetyl-CoA carboxylase (ACC) [124]. Next to insulin, LPS and TNFα as well as the endoplasmic reticulum stress response leads to activation of mature SREBP-1c in the liver. Despite contrary results in humans it seems that SREBP-1c promotes NAFLD progression, as increased expression levels of SREBP-1c and its target enzymes FAS and ACC were determined to be elevated in NAFLD patients [125,126,127,128].

In a murine model of *ob*/*ob* mice suffering from obesity, insulin resistance, hyperinsulinemia and SREBP-1c levels were enhanced [129,130].

Carbohydrate response element-binding protein (ChREBP) is another important transcription factor that controls lipogenesis. In contrast to SREBP-1c, ChREBP is activated by increased glucose levels and is involved in the metabolic change of carbohydrates to TG [131]. Thereby it mediates initiation of transcription of different genes associated with lipogenesis (FAS and ACC), gluconeogenesis (glucose-6-phosphatase) and glycolysis [liver pyruvate kinase (LPK)] [132].

Analysis of different ChREBP knockout mouse models showed less liver steatosis, decreased amounts of SCD1, ACC, FAS and LPK. Moreover, reduction of obesity, insulin resistance and a positive progression of the metabolic syndrome were obtained as well [133,134].

The third transcription factor playing a role in NAFLD is the Liver X Receptor (LXR). LXR in association with retinoid X receptor (RXR) controls the expression of genes involved in cholesterol metabolism, SREBP-1c and ChREBP as well as their target genes ACC and FAS [135,136,137,138,139,140,141]. Metabolites such as glucose and glucose-6-phosphate are activators of LXR.

The role of AMPK in NAFLD is not clearly defined. Generally, AMPK takes part in the energy homeostasis of the cell regarding katabolic processes as fatty acid oxidation and glycolysis and anabolic processes like fatty acid and amino acid synthesis [142]. AMPK has an inhibitory effect on ACC, SREBP-1c and ChREBP77. Experiments with ethanol-fed mice, ethanol treated rat hepatoma cell lines and hepatocytes showed a reduction in AMPK activity correlating with a rising activation of ACC, SREBP-1c and hepatic steatosis progression [143,144]. Further experiments with the AMPK activators metformin or 5-aminoimidazole-4-carboxamide ribonucleoside (AICAR) in rat hepatoma cells and hepatocytes activate AMPK, thereby inhibiting the blocking function of ethanol on ACC and SREBP-1c [143]. Nevertheless, AMPK does not seem to have an important regulatory function in NAFLD progression. Mice fed a high sucrose diet revealed hepatic steatosis, but no differences in the activation level of AMPK were noticed [145]. Moreover, transgenic mice over-expressing constitutively active AMPKα1 in the liver revealed reduced weight gain, white fat mass and blood glucose, which was linked to reduced expression levels of SREBP-1c and its target genes [146]. In a study with rats fed with a HFD for 16 weeks, a reduction of 60% in the activation of AMPK was found. Administration of the AMPK activator Resveratrol for 10 weeks in these animals resulted in less hepatic steatosis and a positive effect on insulin resistance [147].

A further mediator of NAFLD might be a member of the peroxisome proliferator activated receptor family, PPARγ. PPARγ is a nuclear receptor that builds a complex with RXR to promote transcription of its target genes. PPARγ is expressed in a smaller amount in the liver and is predominantly detected in adipocytes. PPARγ is involved in the regulation of adipocyte differentiation, fatty acid uptake and glucose metabolism [77]. Ethanol has the ability to prevent PPARγ expression [148]. PPARγ promotes SREBP-1c activation leading to enhanced expression of lipoprotein lipase in adipocytes [149,150]. Mice with liver-specific PPARγ knockout were reported to rarely develop hepatic steatosis accompanied by hyperlipidemia, triglyceride clearance, and muscle insulin resistance [151]. A study with human patients with a dominant-negative PPARγ mutation revealed that these patients suffered from the MetS and NAFLD [152].

Moreover keratin 8 and 18 was shown to contribute to the development of steatohepatitis [153,154].

## 8. Conclusions

Despite intensive research on steatosis and steatohepatitis, fully satisfying treatment options are currently not available. The best prevention is a life style with balanced nutrition, avoiding excessive alcohol consumption and including sufficient exercise. NALFD/NASH patients suffer from obesity, T2DM and hyperlipidemia promoting the development of fatty liver and increased inflammation resulting in a high susceptibility to develop liver fibrosis, cirrhosis and HCC. Estimation of certain diagnostic parameters revealed a high variability within different human cohorts, which makes it challenging to find a common strategy in diagnosis and treatment.

Work on different murine models led to the identification of different key players, which have a promoting or protecting function in AFLD/ASH or NAFLD/NASH. To which extent they might be translated to the human situation is still questionable. Our expanding knowledge of epigenetics will help us to get a clearer understanding of the molecular mechanisms behind AFLD/ASH and NAFLD/NASH in the near future. Certain studies could show that miRNAs and SNPs might have an important regulatory function in NAFLD/NASH progression.

ASH and NASH show almost identical morphological features. As the clinical presentation is not characteristic, liver biopsy still represents the diagnostic gold standard. Both diseases reveal morphological hallmarks such as steatosis, hepatocellular injury with hepatocytic ballooning, apoptosis, necrosis, inflammation and fibrosis. Differentiation of the two diseases is only possible by confirmation or exclusion of an alcohol abuse. In the case of NASH, obesity is the most constantly associated cause. Ballooning of hepatocytes is linked to a disturbance of the normal keratin-intermediate filament cytoskeleton, being found in both conditions. In some studies on the pathogenic mechanisms of ASH and NASH, altered keratin 8 and 18 could be established as major players for Mallory-Denk body (MDB) formation. In case of an impaired proteolytic activity, protein aggregation can occur [155]. In addition to abstinence from excessive alcohol intake in the case of ASH, experimental models and patients with steatohepatitis have been demonstrated to strongly depend on cholesterol and sphingolipids, in particular on ceramide during the progression from steatosis to steatohepatitis and insulin resistance. Cholesterol accumulation and its transfer to mitochondria render fatty liver more vulnerable to following “hits”, comprising pro-inflammatory cytokines, in a signaling cascade with ceramide generation by acidic sphingomyelinase (ASMase). Therefore, cholesterol and/or ASMase might serve as novel therapeutic targets in ASH and NASH [156].

Support of the multiple-hit hypothesis of NASH is increasing, and the development of new diagnostic techniques is emerging. Therefore the approach to find a list of diagnostic, prognostic as well as predictive parameters, including serum markers, genetic variants, imaging techniques and lifestyle habits will be expanded and promising approaches are on the horizon.
